# Perceptions of pre-clerkship medical students and academic advisors about sleep deprivation and its relationship to academic performance: a cross-sectional perspective from Saudi Arabia

**DOI:** 10.1186/s13104-015-1755-y

**Published:** 2015-12-01

**Authors:** Lama AlFakhri, Jumana Sarraj, Shouq Kherallah, Khulood Kuhail, Akef Obeidat, Ahmed Abu-Zaid

**Affiliations:** College of Medicine, Alfaisal University, Riyadh, 11533 P.O. Box 50927, Saudi Arabia

**Keywords:** Sleep, Sleep duration, Perceptions, Medical students, Academic performance, Saudi Arabia

## Abstract

**Background:**

The medical student population is believed to be at an increased risk for sleep deprivation. Little is known about students’ perceptions towards sleep deprivation and its relationship to academic performance. The aim of study is to explore the perceptions of medical students and their academic advisors about sleep deprivation and its relationship to academic performance.

**Methods:**

The study took place at Alfaisal University, College of Medicine, Riyadh, Saudi Arabia. An online, anonymous, cross-sectional, self-rating survey was administered to first-, third-year students and their academic advisors. Two-tailed Mann–Whitney U test was used to compare the mean 5-point Likert scale responses between students according to gender, academic year and cumulative grade point average (cGPA).

**Results:**

A total of 259 students and 21 academic advisors participated in the survey (response rates: 70.6 and 84 %, respectively). The vast majority of students agreed that sleep deprivation negatively affects academic performance (78.8 %) and mood (78.4 %). Around 62.2 and 73.7 % of students agreed that the demanding medical curriculum and stress of final exams lead to sleep deprivation, respectively. While 36.7 % of students voiced the need for incorporation of curricular separate courses about healthy sleep patterns into medical curriculum, a much greater proportion of students (45.9 %) expressed interest in extracurricular activities about healthy sleep patterns. Interestingly, only 13.5 % of students affirmed that they were counselled about sleep patterns and academic performance by their academic advisors. There were several statistically significant differences of means of students’ perceptions according to gender, academic year and cGPA. Despite almost all academic advisors (95.5 %) asserted the importance of sleep patterns to academic performance, none (0 %) inquired about sleep patterns when counselling students. Nineteen academic advisors (90.5 %) recommended incorporation of sleep patterns related learning into medical curricula; among those, only 1 (n = 1/19; 5.3 %) recommended learning as a separate course whereas the majority (n = 18/19; 94.7 %) recommended learning in forms of extracurricular activities and integration into relevant ongoing courses.

**Conclusions:**

Our results showed that students had correct conceptions about the negative impact of sleep deprivation on academic performance and mood. Also, our results highlighted the need for curricular/extracurricular education and counseling about healthy sleep patterns.

## Background

Sleep is essential for an individual’s growth, health, learning and memory consolidation [1]. Sleep deprivation causes daytime sleepiness, reduced neurocognitive processing and impaired psychomotor performance [2]. Several studies have demonstrated a directly proportional correlation between suboptimal sleep duration (less than 7–8 h of sleep per night) and poor academic performance among college students [3–5].

The medical student population is believed to be at an increased risk for sleep deprivation [6–8]. This can be largely attributed to the altered lifestyles and overloaded academic commitments in order to cope with the demanding medical curriculum [7]. This constant burden, if not properly managed, may lead students to sleep deprivation and successively poor academic performance [9].

Despite students are the principal stakeholders impacted by sleep deprivation in medical schools [6–8], little is known about students’ actual perceptions towards sleep deprivation and its relationship to academic performance. In line with the global tendency towards student-centered education [10], opinions from students provide worthy inputs to medical educators. These inputs are essential in rectifying misconceptions, implementing corrective mechanisms and planning the medical curriculum.

This study has three aims. The first aim is to explore the pre-clerkship students’ overall perceptions towards sleep deprivation and its relationship to academic performance. The second aim is to explore possible differences of the perceptions according to gender, academic year and academic performance—expressed in cumulative grade point average (cGPA). The third aim is to explore the perceptions of academic advisors about sleep and related issues to academic performance, mentee-mentor counselling and curriculum design.

## Methods

### Design and setting

An online, anonymous, cross-sectional, self-rating (5-point Likert scale) study was conducted at Alfaisal University, College of Medicine, Riyadh, Saudi Arabia. The study took place from 19th March 2015 to 16th April 2015 in the spring of 2014–2015 academic year. The College offers a 6-year Bachelor of Medicine, Bachelor of Surgery (MBBS) program. The first 3 years are considered pre-clerkship years. This study was approved by the Institutional Review Board (IRB) at Alfaisal University (IRB Reference Number: 2015-067).

### Participants

The targeted student subjects included all pre-clerkship first- and third-year medical students. The total number of students in the first- and third-year was 367 students. The reason for excluding the second-year students was due to the negligible response rates; they had back-to-back a major end-of-block exam and two parallel courses mid-term exams at the time of study. The targeted academic advisor subjects included all academic advisors mentoring the first- and third-year students. The total number of academic advisors was 25 academic advisors.

### Survey

Students were requested to voluntarily complete an online anonymous survey using the online tool KwikSurveys (Problem Free Company Ltd., Bristol, UK). The survey was administered to explore: (1) medical students’ perceived perceptions towards sleep deprivation and its relationship to academic performance, (2) differences in students’ perceptions according to gender, academic year and cGPA, and (3) academic advisors’ inputs about sleep patterns and its relationship to academic performance.

Sleep deprivation was defined as less than 6 h of sleep per night [11], despite individual variations exist.

**Fig. 1:**
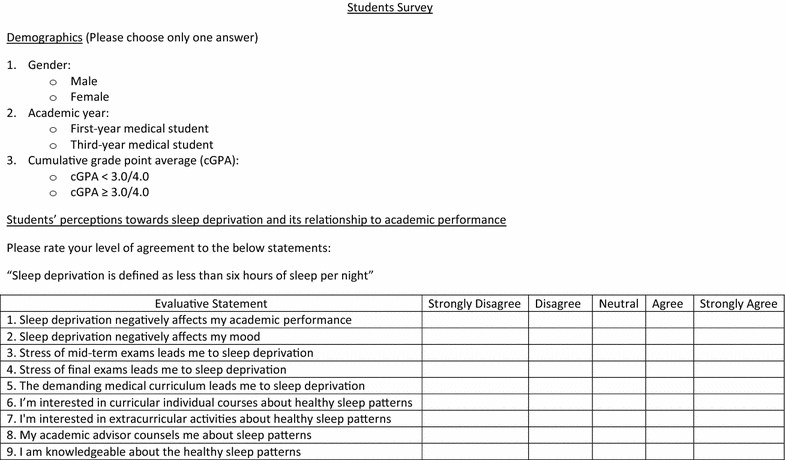
Students survey

**Fig. 2:**
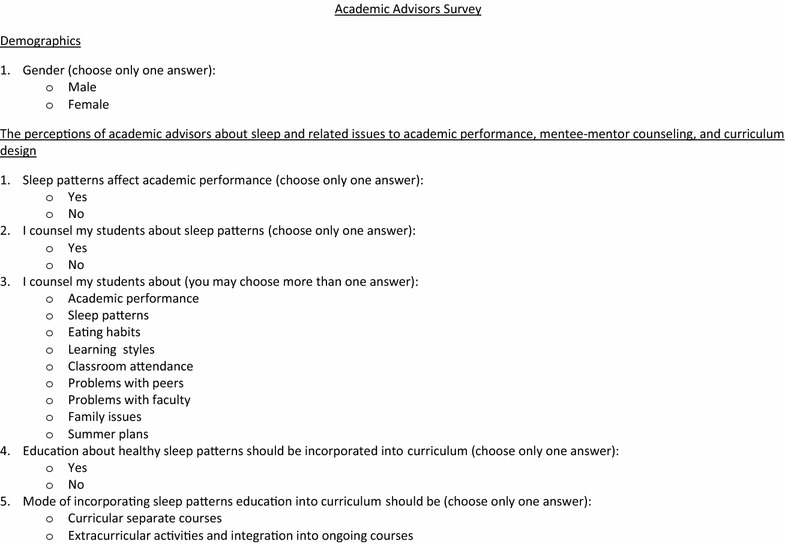
Academic advisors survey

Two surveys were distributed. The first survey (Fig. 1) was distributed to students, and it was constructed partially based on a literature review (that is, published fact) and partially based on newly introduced survey questions that deemed important by the authors. The second survey was distributed to academic advisors (Fig. 2), and it was constructed fully based on newly introduced survey questions that deemed important by the authors. Afterwards, the students survey was peer-reviewed by two external in-house faculty members to verify its proper structure and content. Then it was pre-tested on a set of students (n = 20) to examine its validity and ensure proper interpretation of survey questions. Results of the pre-tested questions looked satisfactory and valid. Also, Cronbach’s alpha coefficient test was used to measure the extent of internal consistency among the tested items. The overall Cronbach’s alpha coefficient was 0.71 indicating acceptable internal reliability of the students survey data.

Students’ characteristics data included: gender, academic year, and self-reported cGPA.

Academic performance was based on the self-reported cGPA, which is a common parameter to determine academic performance in sleep-related research [12, 13]. cGPA was categorized into: “good academic performance/high achievers” (GPA ≥ 3.0/4.0) and “poor academic performance/low achievers” (cGPA < 3.0/4.0). This dichotomous categorization was used in previous research for evaluation of the relationship between sleep and academic performance [12, 13].

The students’ perceptions towards sleep deprivation and its relationship to academic performance were evaluated by the students’ responses to a total of eight typical 5-point Likert rating scale evaluative statements, as follows: (1: strongly disagree, 2: disagree, 3: neutral, 4: agree, and 5: strongly agree). The evaluative statements included perceptions of medical students towards sleep deprivation and its relationship to academic performance and its influential factors, such as: mood, course exam, medical curriculum and academic advisor.

The academic advisors’ inputs about sleep patterns and its relationship to academic performance included questions related to: gender of academic advisor, frequency of counselling students about sleep patterns and the need for incorporation of sleep patterns related education into medical curriculum.

### Statistical analysis

Regarding Likert scale responses, for the purposes of ease in reporting and analyzing data, disagreement responses (1  +  2) were grouped as “disagree”; agreement responses (4  +  5) were grouped as “agree”; and neutral responses (3) were presented as “neutral”. The average 5-point Likert scale responses were presented as means  ±  standard deviations (SD). All calculations of means  ±  SDs for all evaluative survey statements were based on the 5-point Likert rating scale. Categorical data were presented as numbers and percentages. A two-tailed Mann–Whitney U test was used to compare the average 5-point Likert scale responses across gender, academic year and cGPA. For all purposes, statistical significance was determined as a p value <0.05. All data were analyzed using the Statistical Package for Social Sciences version 22.0 (SPSS, Inc., Chicago, IL, USA).

## Results

Two-hundred and fifty-nine students participated in the survey (n = 259/367) with an overall response rate of 70.6 %. There were 117 (45.2 %) male students and 142 (54.8 %) female students. There were 158 (61.0 %) and 101 (39.0 %) first- and third-year students, respectively. According to the dichotomous cGPA categorization, the vast majority of students were high achievers/good academic performance (n = 232/259; 89.6 %) and only a minority were low achievers/poor academic performance (n = 27/259; 10.4 %).

Table 1 shows the students’ overall perceptions towards sleep deprivation and its relationship to academic performance.

**Table 1:** The students’ overall perceptions towards sleep deprivation and its relationship to academic performance

Evaluative statement	All respondents (n = 359)			
	Disagree^a^ n (%)	Neutral^a^ n (%)	Agree^a^ n (%)	Mean ± SD^b^
Sleep deprivation negatively affects my academic performance	18 (6.9)	37 (14.3)	204 (78.8)	4.0 ± 0.9
Sleep deprivation negatively affects my mood	12 (5.0)	43 (16.6)	203 (78.4)	4.1 ± 0.9
Stress of mid-term exams leads me to sleep deprivation	51 (19.7)	65 (25.1)	143 (55.2)	3.5 ± 1.2
Stress of final exams leads me to sleep deprivation	37 (14.3)	31 (12.0)	191 (73.7)	4.0 ± 1.1
The demanding medical curriculum leads me to sleep deprivation	37 (14.3)	61 (23.6)	161 (62.2)	3.7 ± 1.1
I’m interested in curricular individual courses about healthy sleep	52 (20.1)	112 (43.2)	95 (36.7)	3.3 ± 1.1
I’m interested in extracurricular learning activities about healthy sleep	72 (27.8)	68 (26.3)	119 (45.9)	3.3 ± 1.2
My academic advisor counsels me about my sleep patterns	156 (60.2)	68 (26.3)	35 (13.5)	2.3 ± 1.1
I am knowledgeable about the healthy sleep patterns	33 (12.7)	67 (25.9)	159 (61.4)	3.7 ± 1.0

*SD* standard deviation

^a^Disagreement responses (1: strongly disagree, and 2: disagree) were grouped as “Disagree”; agreement responses (4: agree, and 5: strongly agree) were grouped as “Agree”; and, neutral responses (3: neutral) were presented as “Neutral”

^b^The average 5-point Likert scale responses were presented as means  ±  SD; all calculations of means  ±  SDs for all evaluative statements were based on the 5-point Likert rating scale

The vast majority of students agreed that sleep deprivation negatively affects academic performance (78.8 %) and mood (78.4 %). Around 62.2, 55.2, 73.7 % of students agreed that the demanding medical curriculum, stress of mid-term exams and stress of final exams lead to sleep deprivation, respectively. While 36.7 % of students voiced the need for incorporation of curricular separate courses about healthy sleep patterns into medical curriculum, a much greater proportion of students (45.9 %) expressed interest in extracurricular learning activities about healthy sleep patterns. Only 13.5 % of students affirmed that they were counselled about sleep patterns and academic performance by their academic advisors. Around 61.4 % of students stated that they were knowledgeable about healthy sleep patterns.

Table 2 shows the differences of students’ perceptions (self-reported Likert means) towards sleep and its relationship to academic performance according to gender, academic year and cGPA.

**Table 2:** The differences of students’ perceptions (self-reported Likert means) towards sleep deprivation and its relationship to academic performance according to gender, academic year and cGPA

Evaluative statement	Gender				Academic year				cGPA			
	Male (n = 117) Mean (SD)	Female (n = 142) Mean (SD)	U test	p value*	1st-year (n = 158) Mean (SD)	3rd-year (n = 101) Mean (SD)	U test	P value*	High cGPA (n = 232) Mean (SD)	Low cGPA (n = 27) Mean (SD)	U test	P value*
Sleep deprivation negatively affects my academic performance	4.0 (0.9)	4.1 (0.9)	7860	0.45	4.0 (1.0)	4.1 (0.8)	7866	0.85	4.0 (0.9)	4.3 (0.8)	3553	0.25
Sleep deprivation negatively affects my mood	4.0 (0.9)	4.2 (0.9)	7466	0.16	4.0 (1.0)	4.2 (0.8)	7142	0.16	4.1 (0.9)	4.1 (1.0)	3149	0.97
Stress of mid-term exams leads me to sleep deprivation	3.4 (1.2)	3.7 (1.2)	7284	0.09	3.5 (1.2)	3.7 (1.2)	7197	0.18	3.5 (1.2)	4.0 (1.0)	3935	0.03*
Stress of final exams leads me to sleep deprivation	3.9 (1.2)	4.1 (1.1)	7606	0.24	3.9 (1.2)	4.0 (1.1)	7784	0.74	4.9 (1.2)	4.6 (0.6)	4293	0.00*
The demanding medical curriculum leads me to sleep deprivation	3.5 (1.1)	3.9 (1.1)	6761	0.01*	3.6 (1.2)	3.9 (1.0)	6716	0.03*	3.7 (1.1)	4.0 (1.0)	3653	0.16
I’m interested in curricular individual courses about healthy sleep	3.2 (1.0)	3.3 (1.2)	7644	0.27	3.3 (1.1)	3.1 (1.1)	7175	0.17	3.2 (1.1)	3.5 (1.1)	3501	0.32
I’m interested in extracurricular activities about healthy sleep	3.1 (1.2)	3.5 (1.2)	6995	0.03*	3.3 (1.2)	3.2 (1.2)	7635	0.56	3.2 (1.2)	4.0 (1.1)	4307	0.00*
My academic advisor counsels me about my sleep patterns	2.1 (1.1)	2.5 (1.1)	6684	0.01*	2.4 (1.2)	2.2 (1.1)	7183	0.18	2.3 (1.1)	2.4 (1.1)	3290	0.67
I am knowledgeable about the healthy sleep patterns	3.7 (1.1)	3.7 (1.0)	8238	0.91	3.8 (1.0)	3.5 (1.1)	6978	0.09	3.6 (1.0)	4.0 (0.8)	3682	0.14

All calculations of mean (SD) for all evaluative statements were based on the 5-point Likert rating scale

*U test* Mann–Whitney U test score, *SD* standard deviation, *cGPA* cumulative grade point average; high cGPA ≥3.0/4.0; low cGPA <3.0/4.0

^a^A two-tailed Mann–Whitney U test was used to compare the mean 5-point Likert scale responses across gender, academic year and cGPA

^†^Statistical significance, p value <0.05

According to gender, there were statistically significant differences of means between male and female students regarding the following statements: “the demanding medical curriculum leads me to sleep deprivation” (3.5 vs. 3.9, respectively; p < 0.01), “I’m interested in extracurricular learning activities about healthy sleep patterns” (3.1 vs. 3.5, respectively; p < 0.02) and “my academic advisor counsels me about sleep patterns” (2.1 vs. 2.5, respectively; p < 0.01).

According to academic year, there was statistically significant difference of means between first- and third-year students regarding the following statement: “the demanding medical curriculum leads me to sleep deprivation” (3.6 vs. 3.9, respectively; p < 0.01).

According to cGPA, there were statistically significant differences of means between high and low achievers regarding the following statements: “stress of mid-term exams leads me to sleep deprivation” (3.5 vs. 4.0, respectively; p < 0.02), “stress of final exams leads me to sleep deprivation” (3.9 vs. 4.6, respectively; p < 0.00) and “I’m interested in extracurricular learning activities about healthy sleep patterns” (3.2 vs. 4.0, respectively; p < 0.00).

Table 3 shows the perceptions of academic advisors about sleep patterns and academic performance. Twenty-one academic advisors participated in the study (n = 21/25) with a response rate of 84 %. There were 66.7 and 33.3 % male and female academic advisors, respectively. Despite almost all academic advisors (n = 20/21; 95.5 %) asserted the importance of sleep patterns to academic performance, none (0 %) inquired about sleep patterns when counselling students. The most frequent aspects discussed during student counselling were: learning styles (90.5 %), classroom attendance (85.7 %) and summer plans (81 %). Nineteen academic advisors (n = 19/21; 90.5 %) recommended the incorporation of sleep patterns related learning into medical curricula; among those, only 1 (n = 1/19; 5.3 %) recommended introducing it as a separate course whereas the majority (n = 18/19; 94.7 %) recommended learning in the forms of extracurricular activities and integration into relevant ongoing courses.

**Table 3:** The perceptions of academic advisors about sleep and related issues to academic performance, mentee-mentor counselling and curriculum design

	n (%)
Gender (n = 21)	
Male	14 (67.3)
Female	7 (33.3)
Sleep patterns affect academic performance (n = 21)	
Yes	20 (95.5)
No	1 (4.5)
I counsel my students about sleep patterns (n = 21)	
Yes	0 (0)
No	100 (0)
I counsel my students about (n = 21)	
Academic performance	21 (100)
Sleep patterns	0 (0)
Eating habits	3 (14.3)
Learning styles	20 (90.5)
Classroom attendance	18 (85.7)
Problems with peers	4 (19)
Problems with faculty	6 (28.6)
Family issues	12 (57.1)
Summer plans	17 (81.0)
Education about healthy sleep patterns should be incorporated into curriculum (n = 21)	
Yes	19 (90.5)
No	2 (9.5)
Mode of incorporating sleep patterns education into curriculum should be (n = 19)	
Curricular separate courses	1 (5.3)
Extracurricular activities and integration into ongoing courses	18 (94.7)

## Discussion

Our study results are unique in three aspects. First, this study originated from Saudi Arabia (a largely gender-conservative population) which would be of interest to researchers looking at cultural differences. Second, to the best of our knowledge, this is the first study from Saudi Arabia that endeavored to explore the subjective perceptions of medical students towards sleep deprivation and its relationship to academic performance. Third and most importantly, to the best of our knowledge, this is the first study to explore the perceptions of faculty academic advisors about sleep and related issues to academic performance, mentee-mentor counseling, and curriculum design.

Students generally do not recognize the negative impact of sleep deprivation on cognitive functioning and academic performance [14]. In our study, only a few students had misconceptions regarding the negative impact of sleep deprivation on academic performance [9] and mood [14]. Of note, the effect of sleep deprivation on performance was studied in a meta-analysis and results showed that mood was actually more affected by sleep deprivation than either cognitive or motor performance [14]. Therefore, this issue should be critically considered since an individual’s mood is a core determinant of daytime functioning, physical vitality, productivity, decision making, mental sharpness and retention of knowledge.

Striving for excellent academic achievement is one of the top explanations why medical students may modify their total sleep hours per night in college [14]. The most likely modification is sleep deprivation [6–8] in order to meet the continuous pressured academic demands of overloaded curriculum and nerve-racking exams [12].

Female students had higher mean self-reported cGPA than male students (3.7 vs. 3.2, respectively). Sleep deprivation is expected to be higher in female students as they are generally considered high achievers (academically) when compared to their male counterparts [15, 16]; hence, they are more likely to sacrifice hours of sleep to endeavor towards achieving excellent academic performance which is vital to securing postgraduate and employment opportunities.

Furthermore, sleep deprivation is expected to be higher in senior students when compared to junior students as the burden of medical curriculum increases as students dive deeper into medicine and experience the introductory transition to the clinical more demanding education.

Moreover, sleep deprivation is expected to be higher in the low achievers population who possess relatively less cognitive learning skills that likely require them to cut back on total sleep hours in order to meet the demands for additional studying time and better exam preparation. A study has shown that low achievers are likely to expect low grades and hence experience greater degree of stress that simultaneously results in worse sleeping patterns such as sleep deprivation [17]. This will eventually negatively influence exam preparation and performance. Daytime sleepiness is a probable result of sleep deprivation [9, 13]. Daytime sleepiness—as assessed by Epworth Sleepiness Scale (ESS)—was found to be higher in low achieving students than high achieving students in several studies [12, 13].

Students preferred extracurricular over curricular educational interventions to enrich their deficient knowledge about healthy sleep habits. This preference could be attributed to the fact that students do not want to further encumber their already demanding medical curriculum; incorporation of curricular separate (stand-alone) courses would cause an increased burden and study load, which might affect their cGPA negatively. Much emphasis on students’ health related issues, particularly healthy sleep habits, during medical school is greatly needed and should not be neglected. Along the lines of student-centered education, students’ inputs should be valued and honored [10]. A plausible solution to avoid adding separate sleep related courses is a well-designed integration of such learning objectives into ongoing courses such as, physiology, community medicine, public health and family medicine. Furthermore, as suggested by students, extracurricular educational activities about healthy sleep habits should be initiated, in forms of elective courses, interactive workshops, informative on-campus lecture series by experts in the field, and one-to-one dialogues with sleep medicine professionals and others.

It is very worrisome that students are not being counselled about healthy sleeping habits and academic performance by their academic advisors, despite asserting its importance. The unawareness and underestimation of educators of the negative influence of sleep deprivation on cognitive functioning and academic performance has been reported before [14], particularly for the low achieving students. The roles of academic advisors should expand further and refrain from merely discussing learning styles, classroom attendance, problems with individuals, and summer plans to include discussing the often neglected (hidden) issues influencing academic performance; one of which is highlighting the significance of sufficient sleep duration (during weekdays, weekends and before exams) to their academic performance and advancement in medical school [12], particularly for the low achieving students. The Student Affairs departments in medical schools should play key roles in educating their institutions’ academic advisors about healthy sleep habits needed to adequately counsel the medical students.

Several limitations to our study are present. First, this is a self-reported designed study and therefore results are vulnerable to overestimation and/or underestimation by the students. Second, the response rate was less than expected which may have impacted the validity of study. Third, the definition of sleep deprivation variable (less than 6 h per night) was not clearly specified in the survey as whether including workdays and weekends, or most nights or some nights per week/month. It is known that undergraduate students have differences regarding the nocturnal sleep duration in weekdays and weekends.

## Conclusion

In conclusion, our results showed that students had correct conceptions about the negative impact of sleep deprivation on academic performance and mood. Additionally, the study highlighted the need for curricular/extracurricular education and counselling about healthy sleep habits. Future studies are needed to explore other factors leading to sleep deprivation other than the academic environment. Additional future studies include planning and implementing appropriate curricular and extracurricular activities to improve academic performance by targeting the students’ sleep patterns (habits). These are interesting arenas for future research as they are barley explored in literature, particularly in developing countries.

## Abbreviation

cGPA: cumulative grade point average
